# Subjective Cognitive Complaints in individuals with Migraine

**DOI:** 10.1002/alz70857_100176

**Published:** 2025-12-24

**Authors:** Waleska Berrios, Florencia Deschle, Agostina Carra, Juan Pozo, Ana Sanguinetti, Sofia Caporale, Daiana Micuci, Fiorella Martin, Maria Cecilia Fernandez, Guillermo Povedano

**Affiliations:** ^1^ Complejo Médico hospitalario Churruca Visca, buenos aires, Argentina; ^2^ Complejo Médico hospitalario Churruca Visca, Buenos Aires, Argentina; ^3^ Complejo médico hospitalario Churruca Visca, Buenos Aires, Argentina; ^4^ Churruca Visca Hospital, Buenos Aires, Argentina; ^5^ Complejo médico hospitalario Churuca Visca, Buenos Aires, Argentina; ^6^ Hospital Italiano de Buenos Aires, Buenos Aires, Argentina; ^7^ Hospital Italiano de Buenos Aires, Buenos aires, Argentina

## Abstract

**Background:**

Migraine is a highly prevalent and disabling neurological disorder with substantial socioeconomic impact, classified as with/without aura and episodic/chronic based on episode frequency. Chronic migraine patients show greater disability, higher psychiatric comorbidity, and worse neuropsychological outcomes. Cognitive dysfunction, often persisting beyond pain resolution, is a recognized component of migraines. Up to 30% of patients report subjective cognitive complaints (SCC) during the prodromal phase and 38% during the painful phase. SCC refers to perceived cognitive deficits not consistently reflected in neuropsychological testing.

This study aims to compare cognitive complaint reports based on migraine type (with/without aura, episodic/chronic) and levels of disability in migraine patients with normal Montreal Cognitive Assessment (MoCA) scores. Additionally, it analyzes the comorbidity of anxiety and depression.

**Method:**

This cross‐sectional, multicenter study invited patients diagnosed with migraine based on ICHD‐3 criteria. Inclusion criteria: disease duration >1 year, age 30‐60 years, MoCA (Argentinean version) score ≥25. Exclusion criteria: other neuropsychiatric conditions, other chronic pain disorders, or use of cannabis, opioids, or corticosteroids. All participants provided informed consent.

Instruments administered: Argentinean version of Cognitive Complaints Questionnaire (CCQ), Hospital Anxiety and Depression Scale (HADS), and Migraine Disability Assessment (MIDAS) questionnaire.

**Result:**

A total of 42 patients were included: mean age 42.8±9.11 years, mean education 14.6±3.31 years; 88.1% were women. Regarding migraine type: 59.5% had migraines without aura, 71.4% had episodic migraines; mean disease duration 13.7±11.1 years. MIDAS disability levels: no/minimal (35.7%), mild (21.4%), moderate (14.3%), severe (28.6%).

CCQ scores showed no significant differences between patients with aura (24.1±13.1) and without aura (29.1±15.4; *p* = 0.26), or between those with episodic (26.2±14.6) and chronic migraines (29.1±14.9; *p* = 0.57). Similarly, CCQ scores based on MIDAS disability levels were as follows: 20.4±13.6 for no/minimal, 29.9±12.7 for mild, 29.7±17.8 for moderate, and 31.9±13.9 for severe disability (*p* = 0.20).

HADS anxiety and depression subscales showed no significant differences by migraine type or disability levels, but higher scores were observed as disability increased.

**Conclusion:**

Migraine is associated with disability and impacts cognitive and emotional health. No significant differences in SCC were observed based on migraine type or disability levels. The study is ongoing for a deeper understanding of these factors.

